# Beyond the Failure of Direct-Matching in Keyword Evaluation: A Sketch of a Graph Based Solution

**DOI:** 10.3389/frai.2022.801564

**Published:** 2022-03-24

**Authors:** Max Kölbl, Yuki Kyogoku, J. Nathanael Philipp, Michael Richter, Clements Rietdorf, Tariq Yousef

**Affiliations:** Institute of Computer Science, NLP Group, Universität Leipzig, Leipzig, Germany

**Keywords:** keyword evaluation, direct matching, concept formation, word graph, non-symmetric metric

## Abstract

The starting point of this paper is the observation that methods based on the direct match of keywords are inadequate because they do not consider the cognitive ability of concept formation and abstraction. We argue that keyword evaluation needs to be based on a semantic model of language capturing the semantic relatedness of words to satisfy the claim of the human-like ability of concept formation and abstraction and achieve better evaluation results. Evaluation of keywords is difficult since semantic informedness is required for this purpose. This model must be capable of identifying semantic relationships such as synonymy, hypernymy, hyponymy, and location-based abstraction. For example, when gathering texts from online sources, one usually finds a few keywords with each text. Still, these keyword sets are neither complete for the text nor are they in themselves closed, i.e., in most cases, the keywords are a random subset of all possible keywords and not that informative w.r.t. the complete keyword set. Therefore all algorithms based on this cannot achieve good evaluation results and provide good/better keywords or even a complete keyword set for a text. As a solution, we propose a word graph that captures all these semantic relationships for a given language. The problem with the hyponym/hyperonym relationship is that, unlike synonyms, it is not bidirectional. Thus the space of keyword sets requires a metric that is non-symmetric, in other words, a *quasi-metric*. We sketch such a metric that works on our graph. Since it is nearly impossible to obtain such a complete word graph for a language, we propose for the keyword task a simpler graph based on the base text upon which the keyword sets should be evaluated. This reduction is usually sufficient for evaluating keyword sets.

## 1. Introduction

The motivation for the present work is the fact that common keyword evaluation methods, as we will point out below, require an exact match of automatically produced keywords with keywords from a reference or gold standard set. We will argue that this is insufficient modeling of keyword evaluation and propose an evaluation method based on a graph representing the words of a language.

The starting point of a discussion of the evaluation of keywords should clarify the concept, thus what are keywords? (in the following, we use the term *keywords* interchangeably to denote keywords and keyphrases). Çano and Bojar (2019) define keywords as “a short set of one or a few words that represent a concept or a topic covered in a document.” Keywords should fulfil the criterion of “informativeness” (Tomokiyo and Hurst, 2003), i.e., informative parts of a text in alignment with background knowledge. Bharti et al. (2017) follow the definition in Zhang (2008) that keywords reflect the “core sentiment” of a document, and they are utilized for access and recovery of information and documents (Bharti et al., 2017). Due to the descriptive nature of keywords, they are either nouns or noun phrases, i.e., proper names, which was confirmed in previous work from Kölbl et al. (2021). In this paper we maintain this classification. Keywords can be thus regarded as classification features of texts that can be used, among other by search engines. The point of departure is that keyword evaluation raises the problem of comprehension of natural language, which requires a *Common Ground* (CG) of sender and receiver of a message (Karttunen, 1974; Stalnaker, 1974).

A *conditio sine qua non* for successful communication by natural language is an intersection of the shared knowledge in CG: sender and receiver of messages have to dispose over similar mental lexicons, i.e., a sufficiently large intersection of linguistic knowledge of the meaning of words and world knowledge. We claim that (lexical) knowledge in the mental lexicon can be represented by a graph model, where the nodes represent words and the edges represent semantic relations between words. Our approach follows ideas within cognitive psychology, theory of learning, pedagogy and linguistics. Purely conceptual discussion comes from Aebli (1993), and there is massive empirical evidence for modeling the mental lexicon as a graph. The representation of concepts as cognitive units connected within a graph or network (we continue to use the term *graph* in the following) in a mental lexicon (Aitchison, 2012) goes back to Collins and Quillian (1969) (for a modular model, see Fodor, 1983), an assumption, that was empirically underpinned by numerous studies, first by Collins and Loftus (1975), who observed a correlation between the distance of words in a semantic network and the times needed to process those words. This observation was confirmed in more recent studies, amongst others by Dorogovtsev and Mendes (2001), Sigman and Cecchi (2002), and De Deyne et al. (2017), who found that the networks are organized in clusters in order to make distances small that is, to reduce the processing effort (see also Baronchelli et al., 2013; Beckage and Colunga, 2016), and to ensure economic storage (Storkel, 2002; Vitevitch, 2008; De Deyne et al., 2017). Furthermore, the graph model proved to be a powerful model of language acquisition (Storkel, 2002; Carlson et al., 2014; Beckage and Colunga, 2016).

An evaluation of keywords is then based on distances between the nodes representing the lexical units of a language. Consider, for instance, an extracted keyword, like *politician*, while the “true,” or gold standard keyword is its hyponym *Angela Merkel*. In the word graph, there should be only a short distance between *Angela Merkel* and *politician* indicating that they are semantically similar. Consequently, *politician* would not be ruled out a priori (because both chains of letters do not match), rather *politician* would be considered a possible keyword. Furthermore, word pairs like *actress* and *actor* which both have the same meaning up to gender, are interchangeable as keywords since they describe the same concept. We will illustrate the idea of a graph based evaluation with two small example graphs, the words of a fictitious text and a small text from the “Heise” website, respectively. These graphs are manually generated and make no claim to completeness or generalization.

Why is the evaluation of keywords difficult? First, keyword evaluation requires knowledge about the meaning of linguistic units like words, and we postulate that it needs to be based on a semantic model of words capturing how (strong) they are semantically related. This model must be capable of identifying semantic relationships such as synonymy, hypernymy, hyponymy, and a location-based abstraction. It is not a bad choice if, for example, instead of the reference keyword *meeting*, the meaning-similar word *encounter* is generated as a synonym, or if *political system* is generated as a superordinate term, i.e., hyperonym, for *democracy*. The problem with the hyponym/hyperonym relationship is that, unlike synonyms, it is not bidirectional. For instance, a keyword of a text about *Barack Obama, Angela Merkel*, and *Gerhard Schröder* can have *(ex) politicians* as a hyperonym, whereas the keyword *Angela Merkel* as hyponym, i.e., a subordinate term, of *(ex) politicians* cannot be chosen for a text about politicians in general. These semantic relations concern the relation of inclusion in set theory, and philosophic theories about entities and their relations are merologies (Link, 2009). A mereology has a higher level of abstraction than set theory (it abstracts for example from the reduction on rewal numbers; Link, 2009) and is concerned with meronyms and with its opposite concept, the holonym: a meronym is a part of something, for instance is a steering wheel a part of a car, while, *vice versa*, car is a holonym of steering wheel. The space of keyword sets thus requires a metric that is not symmetric, rendering it a *quasi-metric space*. W.r.t. the above example, the distance within the pair 〈politician, Angela Merkel〉 should not equal the distance in 〈Angela Merkel, politician〉.

Second, an evaluation must be able to cope with complex expressions and multiword units, such as *Angela Merkel, Angela Dorothea Merkel, Frau Dr. Angela Dorothea Merkel, the woman formerly known as Angela Dorothea Kasner*, etc. which all refer to the long-term German chancellor. As can easily be seen, the meaning of a multiword expression of that type cannot necessarily be computed following the Fregean *principle of compositionality*. Rather they touch, quite like synonymy, hypernymy, and hyponymy Leibniz's principle of *substitutio salva veritate*: a substitution of a term by another term is possible without changing the truth conditions of the embedding proposition if both terms denote the same entity in the world. This principle is essential in generative summarizations, which make use of generated keywords that do not occur in the source text (see the *Angela Merkel*-*politician*—example from above).

The semantics of proper names in modern philosophy goes back to Leibniz and his principle of *substitutio salva veritate* mentioned above. Frege (1892) and later Kripke (1980) provided counter evidence for this principle, for example in intensional contexts. That is to say, it is not an ontological necessity that a proper name denotes a specific individual, and there is no meaning by definition, or a priori. It was *Cluster Theory* (Strawson, 1950; Searle, 1958) that was introduced as a remedy: the meaning of a proper name is composed from a cluster of attributes of an individual about which there is conventionalized, i.e., general consensus. *Cluster Theory* that has been criticized by Kripke (1980) as possibly none of the attributed characteristics apply to the actual historical individual. Which set of features and which referent are attributed to a proper name is thus essentially dependent on linguistic circumstances, on the conversational context and on individual knowledge of the world. In this paper, we assume that proper names can be keywords. That is to say, for example, that *Prince Charles*, regardless of whom it refers to, can be a keyword of a text, and will in such a case be treated as if it were a single word.

As already briefly stated above, the requirement of an exact match of automatically produced keywords and a reference set neglects the human ability of abstraction and classification (see for instance Aebli, 1993), that is to say, concept formation. Smith (1989) defines a concept as “a mental representation of a class or individual” that has to be distinguished from the external world and thus a concept “[…] deals with what is being represented and how that information is typically used during the categorization.” Goldstone et al. (2017) state that concepts, i.e., mental structures that enable humans to predict categories of entities in the world, are learned inductively. In addition, concepts form the “building blocks of human cognition,” and concept construction is the consequence of the need for “cognitive economy.” This conclusion is supported by information theory: A concept of a category requires fewer bits than storing all members of that category (Goldstone et al., 2017). Consequently, Bruner et al. (1956) states that by building up concepts, the cognitive learning effort is smaller than each instance individually would have to be learned. Thus, to know the name of a concept means to know the hypernym of members of a category, and concepts comprise sets of entities in one category that can be considered linguistically as synonyms, i.e., given a specific context, concepts can be *equivalence classes* (Goldstone et al., 2017). In summary, concept formation can be considered an essential cognitive performance, and we postulate that state-of-the-art methods and techniques of keyword evaluation should be able to approach these skills (Sidman, 1994). However, in previous and recent state-of-the-art studies on keyword evaluation (Hulth, 2003, 2004; Marujo et al., 2015; Tixier et al., 2016), the measures *Precision* (Equation 1), *Recall* (Equation 2), and *F1* (Equation 3) are utilized, where a human-created set of keywords served as a standard (see Section 2).

Another common, not uncontroversial, method that avoids direct matching is the evaluation of keywords by human raters, see for instance (Turney, 2000): there are objections in Hulth (2004) who refers to a report (van Dijk, 1995) on considerable diversities within human ratings. And finally, an extrinsic method of evaluation is, for instance, a task-based evaluation where the generated keywords can be used to accomplish a task faster/better than a baseline approach, as employed in Vijayarajan et al. (2016) for information retrieval in web data. This evaluation method, however, would require an expensive second line of research which would go beyond the scope of this paper.

In the following, we use examples from the German language because it is morphologically more challenging than English, to which *TextRank* (Mihalcea and Tarau, 2004), i.e., a graph-based model for keyword extraction, for example, is tailored (see Section 2). This means that compared to, e.g., English, the greater morphological diversity of German results in a greater number of word tokens and thus in a greater number of possible keyword candidates. For example, in contemporary German, nouns denoting persons almost universally have both a feminine and a masculine form. German also tends to form large compounds and includes extremely complex noun phrases in proper names such as *Bundesanstalt für den Digitalfunk der Behörden und Organizationen mit Sicherheitsaufgaben*, which is the Federal Agency for Public Safety Digital Radio. The morphological richness and word-formation productivity of the German language is intended to underline the problem described below that it is a hard task to form a complete graph of the words of a language.

The structure of the paper is as follows: in Section 2, we sketch previous work on keyword evaluation from different theoretical viewpoints, in 3, the theoretical foundations of keyword sets are given, and in Section 4, we illustrate the structure of the graph. Section 5 defines a quasi-metric for the comparison of a Gold standard keyword set and a set to be evaluated and illustrates the application of this metric by two examples.

## 2. Related Work

As discussed in the introduction, the evaluation method widely used for keyword extraction is Precision, which is the ratio of relevant instances among the retrieved instances (see Equation 1), Recall, the ratio of relevant instances that were retrieved (see Equation 2), and F1, the weighted average of the two (see Equation 3). All three measures are based on direct matching, i.e., the direct comparison of two sets. There are some unique evaluation measures inspired by them or combined with them.


(1) $$\begin{array}{l} \text{Precision}=\frac{\text{true positive}}{\text{true positives}+\text{false positive}} \end{array}$$

(2) $$\begin{array}{l} \text{Recall}=\frac{\text{true positive}}{\text{true positives}+\text{false negatives}} \end{array}$$

(3) $$\begin{array}{l} F_{1}=2\cdot \frac{\text{Precision}\cdot \text{Recall}}{\text{Precision}+\text{Recall}} \end{array}$$

Saga et al. (2014) propose a method named *Topic Coverage* by which the performance of keyword extraction is evaluated without any answer set or reference. The Topic Coverage is defined in Equation (4), where |*E*| denotes the number of elements of set *E*, and *T* is the set of topics in the document sets, which are extracted employing clustering methods such as k-means, etc. Further *E*_*i*_ denotes the set of the top *j* keywords in topic *i*, and *M*_*i*_ is the set of keywords in topic *i* extracted by a certain method to be evaluated. Since this measurement is similar to Recall, the performance of Topic Coverage is examined by the comparison with Recall and is confirmed with their high correlation. In the end, this study concludes that Topic Coverage may be used instead of Recall. Unlike Topic Coverage, our method requires a gold standard keyword set for each text. However, this gives the benefit of being able to judge the quality of a keyword set with a stronger focus on the actual text it was assigned to, instead of having to rely on a topic based average.


(4) $$\begin{array}{l} TC=\frac{1}{\left|T\right|}\underset{i\in T}{\sum }\frac{\left|E_{i}\cap M_{i}\right|}{\left|E_{i}\right|} \end{array}$$

Zesch and Gurevych (2009) use the *R-precision (R-p) measure* for evaluation of keyphrases. They define R-p as the Precision when the number of retrieved keyphrase matchings equals the number of gold standard keyphrases assigned to the document. That is, only extracted keyphrases that are regarded to match the gold standard keyphrases are counted. As for the matching strategy, instead of exact matching, they propose a new approximate matching that accounts for morphological variants (MORPH) and the two cases of overlapping phrases: either the extracted key phrase includes the gold standard keyphrase (INCLUDES) or the extracted key phrase is a part of the gold standard keyphrase (PARTOF). For overlapping phrases, they do not allow character level variations, but only token level variations and morphological variations (MORPH) are limited only to detecting plurals. The evaluation based on these matching strategies is compared to human evaluation, and MORPH matchings put out the best result with 96% agreement to human evaluations. For INCLUDES and PARTOF, on the other hand, agreement to human evaluations is lower. The main difference to our approach is the fact that this method does not take more abstract semantic relationships into account.

Liu et al. (2009) compare the system output to human-annotated keywords using *F-measure*, and in addition to this they also adopt *Pyramid metric* proposed by Nekova and Passonneau (2004). In the Pyramid metric, a score is assigned to each keyword candidate based on how many human annotators selected it. Keywords with a high score are placed at a high level of the pyramid, and the score of hypothesized keywords is computed by adding the scores of keywords that exist in the pyramid. However, since unmatched keywords cannot be measured by these two metrics, they resort to a human evaluation. In this human evaluation, evaluators are asked to exclude non-keywords from the sets of human and machine-generated candidates.

Apart from Precision, Recall and F1, *Pointwise Mutual Information* (PMI) is adopted by Jarmasz and Barrière (2012)'s study for the evaluation of keyphrases. Unlike traditional evaluations based on string matching, the PMI estimates semantic similarity. Thanks to relative scores generated by the PMI, it can be used to compare various keyphrase extraction algorithms.

Graph theory, which has been contributing to various fields of natural language processing, is also indispensable when it comes to evaluation measures. Since the method of the present paper is based on semantic distances in word graphs, it makes sense to consider techniques for *automatic construction of semantic classes* and *identification of semantic distance*.

For automatic construction of semantic classes, the following method is presented by Widdows and Dorow (2002): The method starts by constructing a large graph consisting of all nouns in a large corpus. Each node represents a noun, and two nodes get connected if they co-occur, separated by the conjunctions *and* and *or*. Rare words are filtered out by a cut-off value, that is, the top *n* neighbors of each word, which could be determined by the user. To identify the elements of a semantic class, to begin with, “seed words” as a small set of exemplars are chosen manually. Next, in an iterative process, the “most similar” node is added to the manually selected set of seed words. A candidate node is not added just because of the connection with one single node of the seed set, but rather it is added only when it has a link to some other neighboring node in the seed set. In doing so, the inclusion of an out-of-category word, which happens to co-occur with one of the category words, is avoided. This process is repeated until no new elements can be added to the seed set.

In addition to the automatic construction of semantic classes, the semantic distance between words can be measured given existing semantic networks such as *WordNet* (Miller, 1995; Oram, 2001), in which nouns are organized as nodes into hierarchical structures. Wu and Palmer (1994)'s similarity metric measures what they call *conceptual similarity* between two nodes *c*_1_ and *c*_2_ in a hierarchy (see Equation 5), where depth(*c*_*i*_) is the length of the path to *c*_*i*_ from the *global root*, that is, the top node of the taxonomy. Further lso(*c*_*i*_, *c*_*j*_) denotes the lowest super-ordinate, namely the closest common parent node between *c*_*i*_ and *c*_*j*_.


(5) $$\begin{array}{l} {\mathrm{sim}}_{\text{WuPalmer}}\left(c_{1},c_{2}\right)=\frac{2\mathrm{depth}\left(\mathrm{lso}\left(c_{1},c_{2}\right)\right)}{\mathrm{depth}\left(c_{1}\right)+\mathrm{depth}\left(c_{2}\right)} \end{array}$$

Resnik (1995), using the lso(*c*_*i*_, *c*_*j*_) in combination with information theory, proposes a similarity measure. Let *p*(*c*) be the probability of encountering an *instance* of a concept *c* in the taxonomy such as *WordNet*. For instance, if *c* is “fruit,” its hyponyms such as “apple,” “orange,” etc., are the instances. According to Shannon's information theory, the information content (IC) is −log*p*(*c*), and the semantic similarity between *c*_1_ and *c*_2_ is defined in Equation (6).


(6) $$\begin{array}{l} {\mathrm{sim}}_{\text{Resnik}}\left(c_{1},c_{2}\right)=-\log\text{p}\left(\mathrm{lso}\left(c_{1},c_{2}\right)\right) \end{array}$$

The key idea of this measure is the extent to which two concepts share information in common. If the position of the lowest super-ordinate between *c*_1_ and *c*_2_ is lower, that is, if the closest common parent node of *c*_1_ and *c*_2_ is a less abstract concept, the possibility of encountering an instance of the lowest super-ordinate is lower. That implies a higher IC, which indicates that the two concepts are similar. Moreover, if the lowest super-ordinate of the two nodes is the top node in the taxonomy, their similarity will be −log*p*(1) = 0 (see also Budanitsky and Hirst, 2006).

While it is possible to build our method on top of any of these similarity measures, the constructions we propose are asymmetric. That is because the comparison of a keyword set with a gold standard set is an asymmetric process: if the adequacy of one keyword set implies the adequacy of another, it does not necessarily follow that the same is true the other way around. Hence, we prefer the usage of quasi-metrics rather than metrics to measure semantic similarity.

Nowadays a state-of-the-art method for keyword extraction is the graph-based model, *TextRank* (Mihalcea and Tarau, 2004). In TextRank, text units such as words and sentences are represented as vertices in a graph, and the graph is constructed based on their co-occurrences. In the graph, edges connecting the vertices are defined according to the relation between the text units, e.g., lexical or semantic relations, contextual overlap, etc. As a graph-based ranking algorithm (Mihalcea and Tarau, 2004) modify Google's PageRank developed by Brin and Page (1998) and offer a new formula for graph-based ranking (see Equation 7), where In(*V*_*i*_) denotes the set of vertices pointing to the vertex *V*_*i*_, while Out(*V*_*i*_) denotes the set of vertices that the vertex *V*_*i*_ points to. Further *d* is a damping factor that integrates into the model the probability of jumping from a given vertex to another random vertex in the graph. The damping factor *d* is usually set to 0.85 (Brin and Page, 1998). Next *w*_*ij*_ is defined as a weight of the edge between two vertices *V*_*i*_ and *V*_*j*_. In this regard, it is worth noting that the graph-based ranking in the original PageRank definition is not weighted. In the end, this TextRank algorithm computes scores of the text units by the iteration until convergence and based on the final scores; the relevant text units are extracted. Kölbl et al. (2021) have shown, that TextRank performs very poorly for German texts.


(7) $$\begin{array}{l} WS\left(V_{i}\right)=\left(1-d\right)+d\underset{V_{j}\in \mathrm{In}\left(V_{i}\right)}{\sum }\frac{w_{ji}}{\underset{V_{k}\in \mathrm{Out}\left(V_{j}\right)}{\sum }w_{jk}}WS\left(V_{j}\right) \end{array}$$

Since some lexical ontologies are relevant to our study, brief remarks about them must be made. *WordNet* is the most popular ontology, and nouns, verbs, adjectives, and adverbs are connected with each other based on their semantic relations. The main relation among words in WordNet is synonymy. In addition, the super-subordinate relation such as hypernymy and hyponymy is also integrated. *GermaNet* (Hamp and Feldweg, 1997; Henrich and Hinrichs, 2010) is designed for the German language and shares such common structural features with WordNet. BabelNet (Navigli and Ponzetto, 2012) is a multilingual semantic network constructed from WordNet and Wikipedia. The most distinctive feature of this ontology is that concepts are semantically related to each other across various languages. FrameNet is also one of the lexical ontologies, but it is not constructed based on words *per se*, but on semantic frames (Baker and Fellbaum, 2009).

## 3. Theoretical Foundation

For a text *T* we assume that there exists a complete keyword set *K*_*T*_ that contains all possible keywords for *T*. We can define multiple subsets, firstly we define the subset that contains only keywords that also occur in the text, *K*_*T*, ∩_, and secondly we define the subset that contains all keywords that do not occur in the text, *K*_*T*, \_. As shown in Kölbl et al. (2021) most keywords are names, in most cases either names of persons or organizations, or their abbreviations. This is mostly due to the fact that keywords most often are used in information retrieval systems. Therefore we define a third keyword subset *K*_*T*, names_, which, given the previous, satisfies *K*_*T*, names_⊆*K*_*T*, ∩_. The remaining keyword we will group together in a set, which we will call *K*_*T*, topics_ and which satisfies *K*_*T*, topics_⊆*K*_*T*_\*K*_*T*, names_, mostly contains words which are either topics or abstract descriptive nouns. If *K*_*T*, ∩_ = *K*_*T*, names_ holds then *K*_*T*, \_ = *K*_*T*, topics_.

For example, a text about the German chancellor *Angela Merkel* will have keywords in *K*_*T*, ∩_ like *Angela Merkel, Bundeskanzlerin* (chancellor), or *Merkel* and in *K*_*T*, \_ like *Politik* (politics), *Politikerin* (female politician), or *Angela Dorothea Merkel*. Here we also begin to see depending on the use of a name how random the composition of the sets *K*_*T*, ∩_ and *K*_*T*, \_ is and how it depends entirely on the authors' use. The set *K*_*T*, names_ therefore contains *Angela Merkel* and *Merkel*, both referring to the same person.

**Figure 1:**
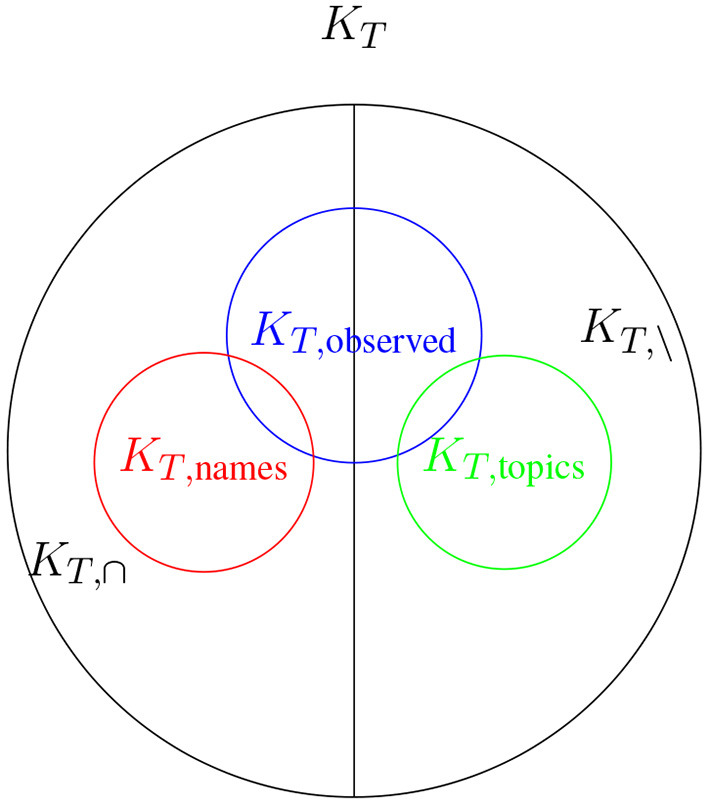
Visualization of all mentioned keyword sets, given that *K*_*T*, names_⊂*K*_*T*, ∩_ and *K*_*T*, topics_⊂*K*_*T*, \_.

Many texts that can be found, e.g., on the internet, have a keyword set associated to them. We call this subset, *K*_*T*, observed_⊂*K*_*T*, ∩_∪*K*_*T*, \_, which is a random subset of some keywords that are in the text and some that are not, and in most cases *K*_*T*, observed_⊂*K*_*T*, names_. For a visualization of all above mentioned keyword sets (see Figure 1).

*K*_*T*, observed_ is the basis of why evaluation methods for keyword extraction/assignment fails. When collecting texts one usually finds the keyword set *K*_*T*, observed_, also known as the *ground truth*. Depending on the praxis of the source of the text, *K*_*T*, observed_ can look very different. For example one online news publication has the mandate to always give four keywords with a text, all of them a topic. Another publication with the mandate to give between three and ten keywords with about half of them occurring in the text. None of them is close to be *K*_*T*_.

Consider the keyword set *K*_*T*,_*P*__*A*__ generated by an algorithm *A*. Let us further assume that *A* returns always all keywords, i.e., *K*_*T*,_*P*__*A*__ = *K*_*T*_. This algorithm will still yield bad Precision, Recall, and F1 values when evaluated against *K*_*T*, observed_. In contrast, when *A* is a perfect named entity recognizer, it will return a superset of *K*_*T*, names_, as not all names need to be keywords, which can be very close to *K*_*T*, observed_ and thus return very good Precision, Recall and F1 values depending. This is the basis for our assumption of why current keyword evaluation methods fail. Approaches based on the direct match between two keyword sets, where one is considered the ground truth, completely rely on the quality of this ground truth set and are unable to account for any abstraction or small differences in the writing, e.g., in a name. This results in three paths forward when it comes to evaluating any algorithm for keyword extraction/assignment: one either can change the evaluation strategy accounting for an imperfect ground truth, meaning that one has to account for words that are closely related to the ones given in the ground truth, or one changes the evaluation strategy completely, no longer requiring ground truth. The third path would ensure that the ground truth always is *K*_*T*_ and not some subset *K*_*T*, observed_, which could be done for some small datasets for a competition or so, but is not feasible for large text corpora. Based on this, we propose a solution along the second lines of the first path, while the approach can easily extended using the word graph to follow the second path.

In the following we consider two examples. First a short toy text that we created to show that through a synonym very unrelated fields are connected. The second text is from an online news site, which is also considerably longer.

Firstly, consider the following text:

(1) *Präsident Obama und Bundeskanzler Schröder trafen sich im Kanzleramt. Sie haben sich unter anderem über Gras unterhalten. Obama hat die Legalisierung während des Abendessens angekündigt*. (President Obama and Chancellor Schröder met in the chancellery. They have talked about weed, among other things. Obama announced the legalization during dinner.)

The keywords are as follows: *K*_*T*, ∩_ = { Präsident Obama (president Obama), Bundeskanzler Schröder (chancellor Schröder), Kanzleramt (chancellery), Gras (grass), Legalisierung (legalization), Obama, Abendessen (dinner)} and *K*_*T*, \_ = { Präsident Barack Obama (president Barack Obama), Barack Obama, Barack Hussein Obama II, Gerhard Fritz Kurt Schröder, Gerhard Schröder, Bundeskanzler Gerhard Schröder (chancellor Gerhard Schröder), Politik (politics), Politiker (politician), Berlin, Bundeskanzleramt (chancellery), Droge (drug), Marihuana (marijuana), Hanf (hemp), THC, Legalisierung (legalization), Schröder, War on Drugs, Drogenkrieg (War on Drugs), Staatsbankett (state banquet), Staatsbesuch (state visit)}. As can be seen, some of the keywords, the names especially, would rarely appear in a news text but often refer to the same thing, i.e., *Präsident Obama, Obama, Präsident Barack Obama* (president Barack Obama), *Barack Obama, Barack Hussein Obama II*, and the author would only pick one of these as a keyword.

Secondly, consider the news article (2) *Atomkraft: Iranisches AKW Buschehr wieder am Netz*^1^ (Nuclear power: Iranian nuclear power plant Bushehr back on the net again) from *Heise online* (a German tech news site), about the Iranian nuclear power plant in Buschehr and its return to power production. Along with the text the following keywords *AKW Buschehr, Atomkraft* (nuclear power), *Iran* are listed, with *AKW Buschehr, Atomkraft* ∈*K*_*T*, ∩_ and *Iran* ∈*K*_*T*, \_.

## 4. Word Graph

Our graph is completely manually constructed, that is to say, it is a (sectional) representation of our mental lexicons, and we created the connections between the nodes according to our intuition. Our evaluation method is based upon a word graph *G* = (*V, E*) that is complete for a given language.

*V* contains a node, for every noun and every proper name, as we want to use the graph for the evaluation of keyword extraction methods, i.e., { Barack, Hussein, Obama, Barack Obama, Barack Hussein Obama II, Bundesanstalt für den Digitalfunk der Behörden und Organizationen mit Sicherheitsaufgaben (Federal Agency for Public Safety Digital Radio)}⊂*V*. For organizations the node has additionally the abbreviation attached, i.e., *BDBOS*, the abbreviation for *Bundesanstalt für den Digitalfunk der Behörden und Organizationen mit Sicherheitsaufgaben (Federal Agency for Public Safety Digital Radio)*. This might also be useful for some nouns, e.g., Atomkraftwerk (nuclear power plant) with AKW, but this is a rather rare occurrence.

Furthermore, since keywords are usually the base form, i.e., the lemma, of a word, nodes *V* contain only this form. The usage of the lemma becomes more important the more tokens a language has. For example, in German, the word *Haus* (house) has the additional forms in the genitive and dative case, respectively, i.e., *Hauses* (house) and *Hause* in singular and in plural in nominative, dative, and accusative case *Häuser* (houses) and in the dative case *Häusern* (houses). Some Slavic languages still have the grammatical number dual, for example in Upper Sorbian the word *dom* (house) has the additional form *doma, domej, domom*, and *domje* in singular, in dual *domaj, domow*, and *domomaj* and in plural *domy, domam, domami*, and *domach*. The usage of the lemma reduces the nodes in the graph significantly, and the usage of some grammatical form has no use as a keyword. In addition, the generic gender forms are used, for instance, *artist* both for a male (Künstler) and a female artist (Künstlerin). In some cases, it may be useful/necessary to have this distinction in the graph or focus on a specific gender. In these cases, it is not a problem to have distinct nodes, but in general the reduction of the number of nodes is more desirable. For a language such as German with a lot of word forms, this has a huge impact on keeping the graph small.

The graph *G* needs to be connected, i.e., there is a path between every pair of nodes. The edges *E* represent different types of relations of the words. There are edges *E* representing synonyms, hypernyms, hyponyms, meronyms, holonyms, location-based abstraction and co-occurrences (could either be sentence co-occurrences or neighborhood). Since many of the relation types are directed, the graph *G* is usually directed. But, if for example only sentence co-occurrences were used to create the edges *E*, the graph would be undirected. In case of a directed graph, every node in *V* needs to have at least an incoming and an outgoing edges, so that in all cases a distance can properly be calculated.

For a word that is a homonym, the corresponding node in *V* has a lot of different edges in *E* representing the different groups of meaning. When considering polysemy, i.e., a linguistic sign, for instance a word, with more than one meaning, where the different meanings have to be related like *mouth* both as part of the human body and as place where a river flows into the sea, there are mainly two approaches: first having a single node and consequently a lot of edges representing the different meanings, or secondly a node for every meaning. Both approaches have their advantages and disadvantages. The first approach requires no knowledge about all the different meanings a word can have. It will implicitly appear in the connection a node has. Whereas for the second approach this knowledge is required when creating the graph and, therefore, is creating the graph more complex. What this means for the metric see the next Section 5. Through the location-based abstraction edges, the graph contains information that, for example, the White House is in Washington, D.C., and thus Washington, D.C. would be a valid keyword for a text about the White House. We assume that this relation is directed.

We also thought about translating the different types of edges as different weights in the graph. This has the advantage that when traversing the graph, some words are closer to another, and would lead to a much more fine-grained distance between the nodes. This would however also mean that when creating the graph, one must decide what weights all these types should have, i.e., is a hypernym relation stronger than a meronym relation? How does this relate to a synonym relation? Since we came to no clear decision here, we decided to use an unweighted graph *G*. Our proposed metric calculates the distance between nodes and thus does not require weighting to be able to calculate the distance. But it might be a good extension to get more fine-grained distances.

**Figure 2:**
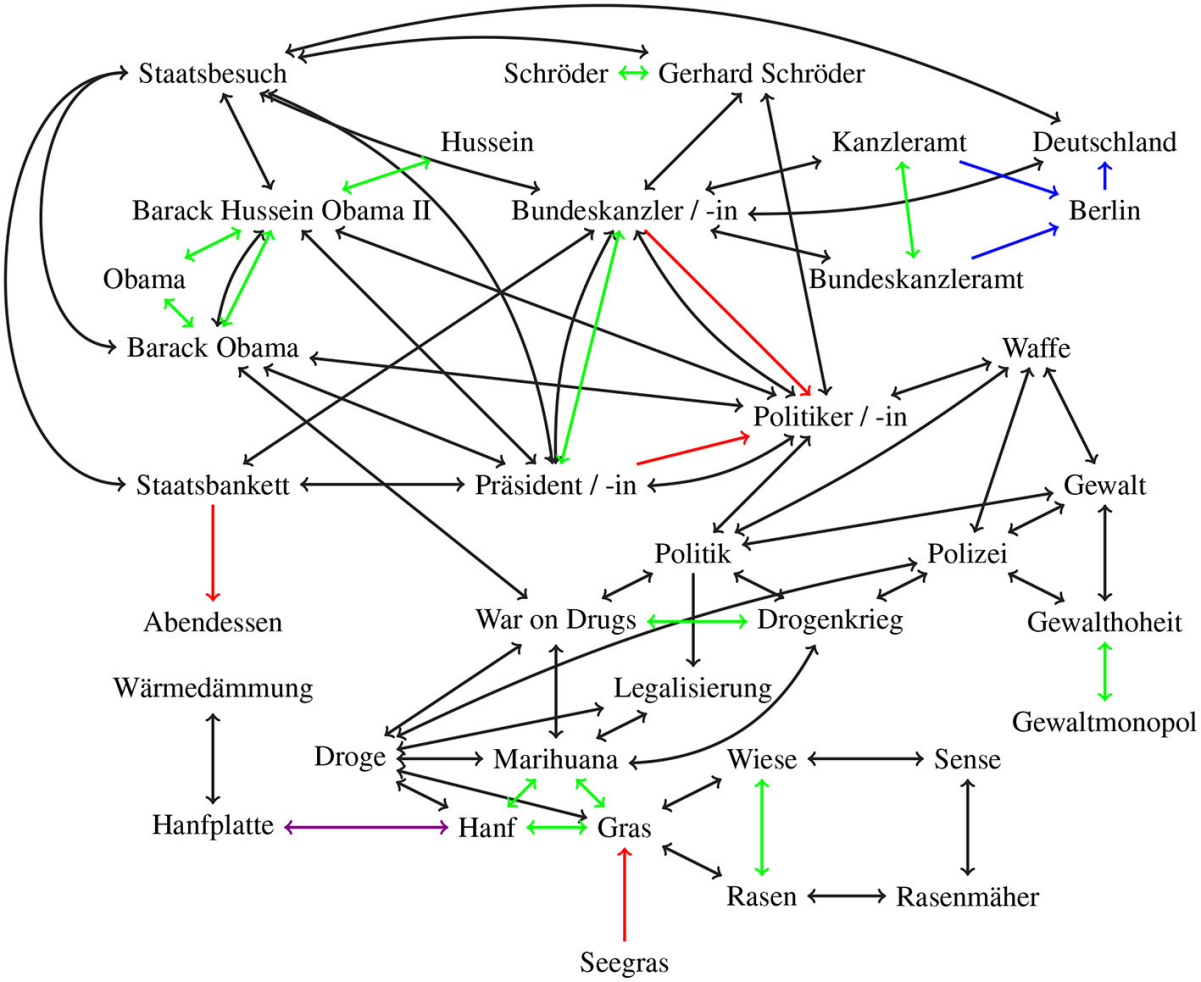
Approximated word graph for our example text. Green arrows represent synonym-relations, red arrows hypernym relations, blue arrows location-based-relation, violet arrows meronym/holonym relations, and black arrows co-occurrences relations. Translations are listed in Table 1.

**Figure 3:**
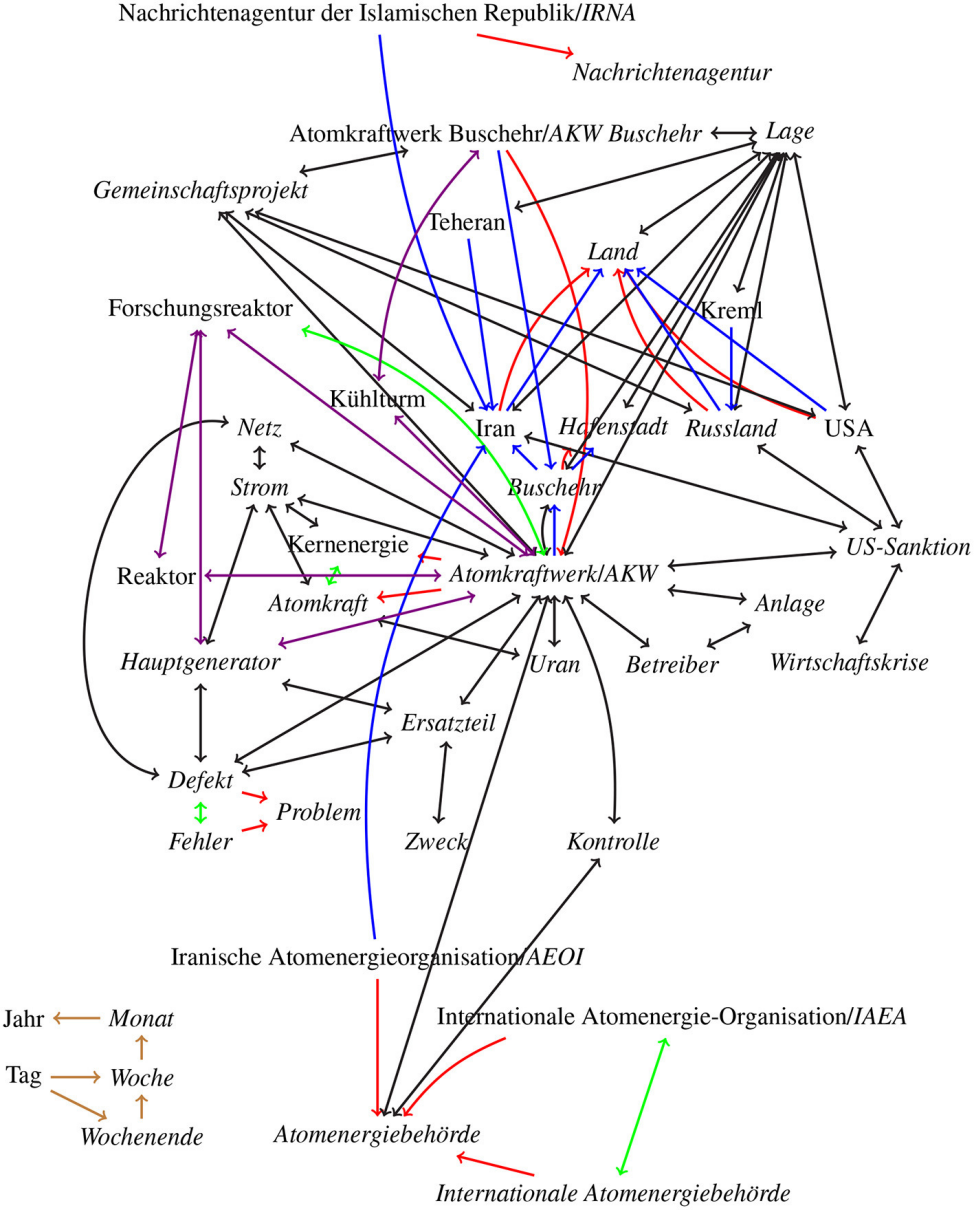
Approximated word graph for our second example text from Heise. All italic words occur in the text. Green arrows represent synonym-relations, red arrows hypernym relations, blue arrows location-based-relation, violet arrows meronym/holonym relations, brown arrows time based-relations, and black arrows co-occurrences relations. Translations are listed in Table 2.

It would be hard to construct such a graph, but it can be approximated. In Figures 2, 3, we show two sections of an approximated graph for our two example texts. We divided the graph into two figures in order to increase clarity and readability. We styled the words in Figure 3 in an italic font that occur in the Heise text. The two figures have several connections, first *Deutschland* (Germany) in the Figure 2 is connected to *Land* (country) in Figure 3, the same way that are *Iran, Russland* (Russia), and *USA* connected to *Land*. Then secondly *Politik* (politics) in Figure 2 is connected to *Atomkraft* (nuclear power), *Kernenergie* (nuclear energy), *Strom* (electricity), and *US-Sanktion* (US sanction) in Figure 3 with black double arrows. And lastly *Präsident/-in* (president) in Figure 2 is connected to *Iran, Russland*, and *USA* in Figure 3, also with black double arrows.

We defined, for simplicity, that *Obama, Hussein, Barack Obama*, and *Barack Hussein Obama II* all refer to the same person and are subsequently synonyms, the same for *Schröder* and *Gerhard Schröder*. In the context of our example text, this is sufficient, but it is not true in general, and different connection types need to be used here. But the impact on the metric and subsequently the keyword sets is irrelevant.

In both graphs, we have included some nodes with multiple connections between each other. One of those double connections is always a co-occurrence relation. This is to demonstrate that almost all words have a co-occurrence relation but that other relations “weigh” more and may thus be preferable. This distinction may be irrelevant when both connections are bidirectional but are otherwise quite relevant.

The creation of such a graph is not trivial. While it is possible to create a graph by hand, it becomes quite inefficient the larger the graph becomes. The two sections in this paper were created by hand, and took quite some time and discussion with the relation to some of the relations. For a larger graph it is therefore desirable to automate this process as much as possible. The easiest method is to just create it from co-occurrences. Here one could use left- and right neighborhood co-occurrences to get directions and use the number of occurrences of a co-occurrence inversely proportional as a weight. Another approach is to use WordNet or rather *GermaNet* for German. While it is a strictly hierarchical graph, it is nevertheless a handcrafted graph of word relations. Some resource as that can be used with some modifications as a basis for a graph.

**Table 1:** Translation for the terms in the Figure 2.

**German**	**English**
Abendessen	Dinner
Bundeskanzler/-in	Chancellor/female chancellor
Bundeskanzleramt	Federal chancellery
Deutschland	Germany
Droge	Drug
Drogenkrieg	War on Drugs
Gewalt	Force/violence
Gewalthoheit	Violence sovereignty
Gewaltmonopol	Monopoly on violence
Gras	Grass
Hanf	Hemp
Hanfplatte	Hemp plate
Kanzleramt	Chancellery
Legalisierung	Legalization
Rasen	Lawn
Rasenmäher	Lawn mower
Seegras	Sea weed
Sense	Scythe
Staatsbankett	State banquet
Staatsbesuch	Statevisit
Politik	Politics
Politiker/-in	Politician (male/female)
Polizei	police
Präsident/-in	President/presidentress
Waffe	Weapon
Wärmedämmung	Thermal insulation
Wiese	Meadow

**Table 2:** Translation for the terms in the Figure 3.

**German**	**English**
Anlage	Plant, installation
Atomenergiebehörde	Atomic energy agency
Atomkraft	Nuclear power
Atomkraftwerk/AKW	Nuclear power plant
Betreiber	Operator
Defekt	Malfunction
Ersatzteil	Spare part
Fehler	Error, mistake, bug
Forschungsreaktor	Research reactor
Gemeinschaftsprojekt	Joint project, partnership
Hafenstadt	Port city
Hauptgenerator	Main generator
Internationale Atomenergiebehörde	International Atomic Energy Agency
Internationale Atomenergie-Organization	International Atomic Energy Agency
Iran	Iran
Iranische Atomenergieorganization	Atomic Energy Organization of Iran
Kernenergie	Nuclear energy
Kontrolle	Control, inspection
Kreml	Kremlin
Kühlturm	Cooling tower
Lage	Location, position
Land	Country, land
Monat	Month
Nachrichtenagentur	News agency
Nachrichtenagentur der Islamischen Republik	Islamic Republic News Agency
Netz	Network
Problem	Problem
Reaktor	Recator
Russland	Russia
Strom	Electricity
Uran	Uranium
US-Sanktion	US sanction
Wirtschaftskrise	Economic crisis
Woche	Week
Wochenende	Week end
Zweck	Purpose

## 5. Comparing Sets of Keywords

For a text *T*, we now want to find a way to compare the set of given keywords *K*_*T*, observed_ with the set *K*_*T, A*_, which is a set of keywords given by some algorithm *A*. This means of comparison ought to be based on the “semantic distances” between the keywords of the two sets. Intuitively, it is supposed to measure how much sense it makes to substitute a given non-empty set of keywords *K*_1_ by the non-empty set *K*_2_. In other words, we have a function μ_*sd*_(*K*_1_, *K*_2_) which returns a non-negative real number. The subscript *sd* stands for the semantic distance function *sd*(*w*_1_, *w*_2_) between a word *w*_1_ and a word *w*_2_. The higher the number, the larger is the semantic difference between the sets. The basic assumption for this function is that *K*_1_ is an already perfect set of keywords, and *K*_2_ needs to be as semantically close as possible. Hence, if we want to add new keywords, we are concerned with how well they will fit in. A keyword set will typically consist of words throughout a greater range of topics. However, since μ_*sd*_ cannot consult the text to see whether it is sensible or not to add a semantically distant keyword, we want to stay on topic. This motivates the first condition.


(M1) $$\begin{array}{l} \mu_{sd}\left(K_{1},K_{2}\right)=\frac{\underset{w_{2}\in K_{2}}{\sum }\underset{w_{1}\in K_{1}}{\min}sd\left(w_{1},w_{2}\right)}{\left|K_{1}\cup K_{2}\right|}\text{if }K_{1}\subseteq K_{2} \end{array}$$

If we want to take keywords away, we want to avoid losing as many semantically distant words as possible because they likely represent different topics in the text. Hence, we assume that taking keywords away is a means to get rid of redundancies. This justifies the second condition.


(M2) $$\begin{array}{l} \mu_{sd}\left(K_{1},K_{2}\right)=\frac{\underset{w_{1}\in K_{1}}{\sum }\underset{w_{2}\in K_{2}}{\min}sd\left(w_{1},w_{2}\right)}{\left|K_{1}\cup K_{2}\right|}\text{if }K_{1}⊇K_{2} \end{array}$$

Now we can define μ_*sd*_ for any two non-empty sets of keywords as follows by combining (M1) and (M2).


(M3) $$\begin{array}{l} \mu_{sd}\left(K_{1},K_{2}\right)=\mu_{sd}\left(K_{1},K_{1}\cup K_{2}\right)+\mu_{sd}\left(K_{1}\cup K_{2},K_{2}\right) \end{array}$$

With these conditions, substituting a keyword set *K*_1_ by another set *K*_2_ first and then substituting *K*_2_ by a third set *K*_3_ cannot yield a better result than substituting *K*_1_ by *K*_3_ directly. Hence, μ satisfies the triangle inequality. On the other hand, if a keyword set *K*_1_ were to be substituted by itself, both (M1) and (M2) [and hence (M3)] evaluate to 0, which means that μ satisfies the identity axiom of metrics.

Thus, Equations (M1) to (M3) almost fit the definition of a metric. Only symmetry is missing, but in general, we do not want that. For instance, consider the example text from the second section. If we take a keyword set *K*_1_ containing the word *Gras* “*weed*,” for this specific text, whose context is drug legalization, it would be unreasonable to substitute *Gras* for, e.g., *Seegras* “*sea weed*.” However, if the situation was reversed and *Seegras* was given in a keyword set, substituting it for the more general word *Gras* is reasonable. In practice, this may not be an issue, and μ_*sd*_ might become a de-facto-metric with respect to the possible keywords of a given text, but for all possible texts, this cannot be assumed. Hence, μ_*sd*_ is only a quasi-metric. For further information on quasi-metrics (see Wilson, 1931).

Given a text *T* with a set of possible keywords *K*_*T*_, we want to define μ_*T*_: = μ_*s*_*d*__*T*__ *via* the word graph from the previous section. Consider the graph *G*_*T*_ = (*V*_*T*_, *E*_*T*_) with *V*_*T*_ = *K*_*T*_ and *E*_*T*_ being the set of edges $\left(w_{1},w_{2}\right)\in K_{T}^{2}$ which lie also in the word graph. The most straightforward definition of *sd*_*T*_(*w*_1_, *w*_2_), and hence of μ_*T*_, is as the length of the shortest path from *w*_1_ to *w*_2_ or a multiple thereof. The following example provides some evidence as to why this is a choice.

Assume the example text from Section 3 has the following keywords: *Barack Obama, Gerhard Schröder, Legalisierung* (legalization), *Droge* (drug). An algorithm choosing words from *K*_*T*, ∩_ might reasonably return the keywords *Obama, Schröder, Gras* (weed), which is arguably not a bad choice. However, with all the “correct” keywords lying in *K*_*T*, \_, there is no intersection, which means that Precision, Recall and F1 would all be 0.

Their distance under *sd*_*T*_ is 8 and μ_*T*_ is 8/7 = 1.143, because, firstly, the distance between the sets {Barack Obama, Gerhard Schröder, Legalisierung, Droge} and { Barack Obama, Gerhard Schröder, Legalisierung, Droge, Schröder, Obama, Gras } is 3/7 (we iterate over the words in the second set. *Barack Obama, Gerhard Schröder, Legalisierung*, and *Droge* all appear in the first set as well, so their distances with the closest words in the other set are all 0. The remaining words, *Obama, Schröder*, and *Gras*, have minimal paths, respectively, starting at *Barack Obama, Gerhard Schröder*, and *Droge*, each with distance 1) and {Schröder, Obama, Gras} is 5/7 (we iterate over the words in the first set. *Obama, Schröder*, and *Gras* all appear in the second set as well, so their distances with the closest words in the other set are all 0. The remaining words, *Barack Obama, Gerhard Schröder, Legalisierung*, and *Droge*, have minimal paths, respectively, starting at *Obama, Schröder, Gras*, and *Gras* again, each with distance 1 except for the path from *Legalisierung* which has length 2). This is not bad for keyword sets of these sizes and very good considering that they do not intersect (the lowest possible distance there is 1). Compared to Precision, Recall and F1, our approach shows a clear superiority in capturing the semantic nearness of *K*_*T*, \_ and *K*_*T*, ∩_. In comparison, a nonsensical keyword set of the same size, say, *Waffe* (weapon), *Berlin, Rasenmäher* (lawn mower) would achieve a distance of 17 and μ_*T*_ = 2.429.

In keyword sets that intersect, the distance will yield lower values. Take for example the set *Barack Obama, Gerhard Schröder, Staatsbesuch* (state visit) (resulting in a Precision, Recall and F1 of 0.667). Even though two keywords are identical, the metric gives a slightly higher value of μ_*T*_ = 1.2, due to the distances of *Droge* and *Legalisierung*. Since the semantic aspect of drug legalization gets lost entirely, the value is still relatively high.

In our second, “real world,” example text *Atomkraft: Iranisches AKW Buschehr wieder am Netz* from Heise, as mentioned in Section 3 above, the given keyword set is *K*_*T*, observed_ = { AKW Buschehr, Atomkraft, Iran }. We compare this with the set *K*_1_ = { Atomkraftwerk, Kernenergie, Kühlturm, Forschungsreaktor, Teheran, Kreml }. Except for *Atomkraftwerk* all other keywords in *K*_1_ are not in the text and both keyword sets have no keyword in common, resulting in a Precision, Recall and F1 of 0. The distance between the two keyword sets is μ(*K*_*T*, observed_, *K*_1_) = 1.22. Once again, this illustrates the advantage of our approach: *K*_*T*, observed_ and *K*_1_ do not intersect and consequently do not match directly, but, intuitively, *K*_1_ is not a bad set of keywords for the text, and our approach manages to express this intuition.

In the case of polysemy, as mentioned in the previous Section 4, there are two cases to consider. In the first case, when each meaning has its own node, consider the following: with a keyword set *K*_1_ with words *w*_1_ and *w*_2_, *K*_1_ = {*w*_1_, *w*_2_}, where there are two nodes *n*_*w*_1_, 1_ and *n*_*w*_1_, 2_ for *w*_1_ and one node for *w*_2_ in the graph *G*_1_. When *K*_1_ with some other keyword set *K*_*o*_, μ_*sd*_(*K*_1_, *K*_*o*_) is compared, the node for *w*_1_ needs to be resolved before calculating *sd*(*w*_1_, *w*_*i*_) with *w*_*i*_∈*K*_*o*_. This is to say, $\forall w\in K_{1}\backslash w_{1}:\underset{\left\{n_{w_{1},1},n_{w_{1},2}\right\}}{\min}sd\left(w_{1},w\right)$, the closest node to the other words in the keyword set must be found which is then used for the comparison. This obliviously falls short when there is only one keyword with multiple meanings in one of the keyword sets that should be compared. If there are multiple words in a keyword set with multiple meanings this gets significantly more complex, but should result in a minimum.

In the second case there is a single node for each word *w*, regardless of how many meanings there are. The graph has therefore nodes that connect some node clusters with very different meanings. In the cases of the example graph in Figure 2 this is the word *Gras*, that connects the drug related nodes to gardening related node. Comparing two keyword sets *K*_1_ and *K*_2_, μ_*sd*_(*K*_1_, *K*_*o*_) in this case it is significantly simpler to calculate *sd* since there is always only one node for each word, but the distances are much smaller. Even with a cross comparison between all words in the two keyword sets it might not be possible to identify all wrong keywords. In the case of the mentioned word *Gras* consider the two keyword sets *K*_1_ = { Gras, Droge } and *K*_2_ = { Gras, Rasen }, the distances are as follows *sd*(Gras, Gras) = 0, *sd*(Gras, Rasen) = 1, *sd*(Droge, Gras) = 1, and *sd*(Droge, Rasen) = 2. This result might be leading to the wrong conclusion, that these two sets are very good keyword sets for our text.

While our proposed method works only if there is some kind of ground truth keyword set, which is a somewhat limiting factor, an argument can be made that if there is no ground truth keyword set available the text itself could be used. For such an approach to work one needs to extract all nouns/names from the text, lemmatize them and use them as a ground truth keyword set (*K*_*T, N*_). While this is far from a good keyword set, we know that *K*_*T*, ∩_⊆*K*_*T, N*_ holds. The distance in the graph and the resulting value of the metric should best be especially low to be considered a good keyword set.

## 6. Conclusion

Popular keyword evaluation methods rely on direct matching without any regard to semantic nuance, making them fast to assign a low level of accuracy to a perfectly adequate keyword set. Hence, we propose using a word graph to provide a richer semantic structure that an evaluation method can use to cast more refined judgment. The advantage becomes clear when comparing our approach with a Precion-Recall-F1-based evaluation: the latter evaluated intuitively good keyword sets, when compared to gold standard sets, as completely deviating and non-fitting. In contrast, our approach, albeit illustrated only by two small examplary and intuitively generated graphs, showed the semantic closeness of the sets to be evaluated and gold standard sets. Since the construction of a complete word graph is an extremely hard task for a language, however, finding manageable, text-specific approximations without sacrificing too much of their quality would presumably fulfil the task with satisfactory results. This may prove difficult enough already: recall that even the graph of our simple three-sentence-example-text is quite extensive and complex despite only being a sample.

Since keywords still have to be topical, it makes sense to only approximate the graph locally, i.e., around the text whose keyword set is to be checked. Given a text *T*, the most radical local approximation is the graph *G*_*T*_, which only uses the words in *T*. This would, however, limit us only to find keywords of the set *K*_*T*, ∩_. The task now is to extend *G*_*T*_ by a reasonable amount to include words related to the words in *G*_*T*_ (whatever that means). Finding a good way to do so is not a trivial task either.

Hence, our focus for further research is to try and test different extension paradigms. For instance, using resources such as WordNet (Miller, 1995) or in the instance of German *GermaNet*, extending *G*_*T*_ by all hyperonyms to $G_{T}^{\text{hyper}}$ might be a good place to start. Suppose a text contains the keyword *Angela Merkel* but not the word *Politiker* “politician” (which is a hyperonym). In that case, the latter is still a valid keyword and would be included in the graph $G_{T}^{\text{hyper}}$. The reverse is not generally true.

Basing $G_{T}^{\text{hyper}}$ on *WordNet* also opens up the possibility to use, for example, the information theory based metric defined in Resnik (1995) to measure the semantic distance between concepts and words. Another possibility, also based on information theory, is that given a specific context, say a text, a gold standard set of keywords and a keyword set to be evaluated should be similarly informative: the information distance (=difference) between both sets of keywords should be small, and a word in the keyword set to be evaluated should be similarly informative to its counterpart in the gold standard set.

A small distance in information would thus represent a small distance in meaning, and an ideal set to be evaluated would have a distance of 0 to the gold standard set. But that as well is likely to prove infeasible for a keyword evaluation algorithm. Hence, once a decent approximation has been found, another aim is to construct a fast heuristic, for example, to train a neural net or another statistical model with graph data.

Finally, we would like to stress the following point: the determination of hierarchy relations within the graph are theory and model dependent and based on techniques of epistemology. A graph can be—based on corpus data—automatically generated or, alternatively, based on judgements of raters. Automatic generation of graphs is often based on statistical regularities of co-occurrences of words. Semantic similarity of two words can be represented through a similar context, which is the view in a distributional theoretic framework. But this depends on the size and quality of the data basis of a study, so that an automatically generated graph will sometimes show semantically implausible relations between words, and semantic relations such as hyperonymy, hyponymy, meronymy, etc. can not be captured. Our graph is a (sectional) representation of individual mental lexicons because the strength of the semantic relations between nodes in the graph, i.e., the words, their hierarchy (synonymy, hyperonymy, hyponymy) was determined by our intuition. In cognitive psychology and theory of learning, it depends on the individual experience of the language learner how relations in the world are structured in cognition, however, it seems to be indisputable that knowledge is organized by abstraction into concepts, i.e., semantic fields or classes that are structured by semantic relations (Aebli, 1993) as, in principle, represented by the graphs in this work.

## Data Availability Statement

The original contributions presented in the study are included in the article/supplementary material, further inquiries can be directed to the corresponding author/s.

## Author Contributions

All authors contributed equally, making the concepts, and writing the article. All authors contributed to the article and approved the submitted version.

## Conflict of Interest

The authors declare that the research was conducted in the absence of any commercial or financial relationships that could be construed as a potential conflict of interest.

## Publisher's Note

All claims expressed in this article are solely those of the authors and do not necessarily represent those of their affiliated organizations, or those of the publisher, the editors and the reviewers. Any product that may be evaluated in this article, or claim that may be made by its manufacturer, is not guaranteed or endorsed by the publisher.

## Footnotes

^1^ https://heise.de/-6128524
